# Age-related differences in the weighting of kinematic and contextual information during action prediction

**DOI:** 10.3389/fnagi.2025.1622569

**Published:** 2025-08-18

**Authors:** Elisa Ravizzotti, Alessandra Finisguerra, Gaia Bonassi, Carola Cosentino, Susanna Mezzarobba, Alessandro Botta, Martina Putzolu, Sara Terranova, Valentina Bianco, Laura Avanzino, Elisa Pelosin, Cosimo Urgesi

**Affiliations:** ^1^Department of Neuroscience, Rehabilitation, Ophthalmology, Genetics and Maternal Child Health (DINOGMI), University of Genoa, Genoa, Italy; ^2^Scientific Institute, IRCCS E. Medea, Pasian di Prato, Udine, Italy; ^3^IRCCS Ospedale Policlinico San Martino, Genoa, Italy; ^4^Department of Experimental Medicine (DIMES), Section of Human Physiology, University of Genoa, Genoa, Italy; ^5^Department of Brain and Behavioral Sciences, University of Pavia, Pavia, Italy; ^6^Laboratory of Cognitive Neuroscience, Department of Languages and Literatures, Communication, Education and Society, University of Udine, Udine, Italy

**Keywords:** action prediction, action observation, aging, social perception, context

## Abstract

**Introduction:**

Predicting others’ behaviors is an essential ability to interact efficiently within the social world. Previous evidence suggests that action prediction entails the integration of incoming sensory information with previous experience and contextual expectations. While it is well known that motor and cognitive functions face age-related changes, research examining how action prediction abilities evolve across the lifespan remains limited.

**Methods:**

Here, we compared the action prediction performance of 30 young and 30 older adults in a temporal occlusion paradigm displaying everyday actions embedded in breakfast scenarios. We asked participants to predict the outcome (i.e., to eat or to move) of reaching-to-grasp movements towards big or small food objects (i.e., krapfen or cream puff). Actions were embedded in contexts cueing to an eating or a moving intention, either congruently or incongruently with kinematics. We also measured participants’ imaginary abilities and level of identification of actions.

**Results:**

Compared to young adults, older adults showed lower sensitivity at predicting actions when they were interrupted early, but not later. At the same time, they were less affected by response bias, particularly for late-interrupted actions. Beside reduced sensitivity, older adults’ response speed in predicting early-interrupted actions benefitted more than that of young adults from contextual information. Notably, contextual modulation was stronger in individuals with more intense kinesthetic sensations during motor imagery, particularly within the young group.

**Discussion:**

The results suggest that, while action prediction skills seem to reduce with aging, older adults tend to rely more heavily on contextual cues when predicting others’ behavior, which may serve as a compensatory mechanism under certain conditions.

## 1 Introduction

Action prediction is a fundamental cognitive ability in the realm of human perception and social interaction and underpins our capacity to navigate the dynamic world around us (Blakemore and Decety, 2001; Spiers and Maguire, 2006) by anticipating and adapting to the actions of others (Knoblich and Flach, 2001) enabling efficient and contextually appropriate responses (Bubic, 2010). This predictive process permeates various facets of our daily lives, from understanding the intentions of a friend’s subtle gestures to safely maneuvering through a bustling city intersection.

Action prediction not only plays a key role in social and physical interactions, but it also participates in higher-order cognitive processes such as decision-making, learning and motor control (Brown and Brüne, 2012). Indeed, it involves the capacity to extrapolate future events, often in real-time, based on a set of available sensory cues, prior experiences, and probabilistic inferences (Bianco et al., 2022; Graf et al., 2007; Springer et al., 2011).

According to the predictive coding framework (Clark, 2013; Friston, 2005), during action perception, incoming sensory signals are constantly matched with internally generated predictions which rely on prior observers’ knowledge and expectations (Friston, 2012; Kilner et al., 2007). Thus, predictions emerge through a dynamic combination of bottom-up sensory inputs and a top-down internal forward model enriched by previous experiences (Teufel and Fletcher, 2020) involving a real-time simulation (Graf et al., 2007; Springer et al., 2011), and reinforced by contextual explicit feedback (Bianco et al., 2022). This is achieved through a hierarchical process in which higher-level brain regions generate top-down predictions that are sent to lower-level regions. Meanwhile, bottom-up sensory inputs are processed and compared with these predictions. If there is a mismatch, prediction errors are sent back up the hierarchy to update the predictions and refine the internal model.

This predictive framework guides us in deciphering the intentions, goals, and likely courses of action of individuals in our immediate environment. It enables us to make split-second decisions, anticipate potential outcomes, and formulate accurate motor responses, all of which are crucial to fluidly navigating complex social and physical landscapes. Consistent with this, previous research has shown that observers’ action prediction performance, as well as their motor activation during action observation, is facilitated when action kinematics (such as speed, direction, and motion patterns), unfolds within contextual settings that point to a congruent compared to an incongruent intention (Amoruso et al., 2016, 2018, 2020; Amoruso and Urgesi, 2016; Betti et al., 2022; Bianco et al., 2024).

In addition to sensory and contextual processing, motor imagery, defined as the ability to internally simulate actions without overt movement (Decety, 1996), and action identification, defined as the observer’s ability to access and label the intention behind an action (Blakemore and Decety, 2001; Spiers and Maguire, 2006), are considered relevant mechanism for action prediction. Higher motor imagery vividness has been associated with enhanced ability to anticipate others’ actions (Lewkowicz et al., 2013; Springer et al., 2012). Similarly, action identification may improve predictive accuracy, particularly in situations where kinematic or contextual information is ambiguous or conflicting (Bianco et al., 2024; Cavallo et al., 2016; Stapel et al., 2015).

So far, research on action prediction has primarily focused on young healthy subjects (Decroix and Kalénine, 2019; Pesquita et al., 2016) and athletes (Makris and Urgesi, 2015; Mulligan and Hodges, 2014; Ottoboni et al., 2021; Urgesi et al., 2012), with limited exploration of age-related changes. While some behavioral studies suggest that action prediction may decline with age, conflicting evidence exists. For instance, Diersch et al. (2012, 2013) demonstrated that older adults exhibited lower accuracy and perceptual sensitivity in predicting the time course of observed actions compared to young adults, although performance was influenced by previous experience with the observed actions. Differently, Sacheli et al. (2023)found no difference between older and young people in terms of sensitivity and efficiency during an action prediction task.

Neurophysiological findings indicate more robust age-related activation changes, particularly in the Action Observation Network (AON). The AON comprises cortical regions, including premotor, inferior frontal, parietal, superior temporal sulcus, and occipito-temporal areas, and subcortical structures, such as cerebellum, caudate and subthalamic nuclei, globus pallidus, and thalamus. These brain regions are engaged in representing both low-level (kinematics) and high-level (goals, and intentions and expectation) aspects of observed actions (Balser et al., 2014; Buccino et al., 2001; Diersch et al., 2013; Downing et al., 2001; Errante and Fogassi, 2020).

AON activity in the human brain is modulated according to the purpose of the observed action, with varied cortical responses depending on whether the movement involves interaction with an object or with another person (Becchio et al., 2024; Centelles et al., 2011). Analyzing AON functionality, research revealed a hypoactivation of posterior parietal regions (Sacheli et al., 2023) and caudate (Diersch et al., 2012, 2013, 2016) in older compared to younger adults during a prediction task. Furthermore, older individuals showed increased activation in the visual cortex and in the medial orbitofrontal cortex, partly reflecting less specific sensory representations of the observed actions (Baltes and Lindenberger, 1997), depending on the familiarity with the observed actions (Diersch et al., 2013).

Previous studies that have examined age-related differences in visual object perception have shown that aging is associated with decreased weighting of sensory information and greater reliance on top-down influences (Chan et al., 2021; Gilbert and Moran, 2016; Hsu et al., 2021; Trapp et al., 2022). In particular, studies have shown that older adults are more affected than young adults by the context in which objects are embedded while categorizing (Rémy et al., 2013) or identifying (Lai et al., 2020) objects. Still, we are unaware of previous studies of aging-related changes of context-based action predictions.

In sum, the actual understanding of action prediction has predominantly centered around young healthy individuals and athletes, leaving age-related changes relatively unexplored with few, contradictory pieces of evidence. This highlights the need for further investigation.

Given this gap in research, our study aimed to delve deeper into age-related differences in action prediction when observing everyday actions, using a temporal occlusion paradigm. Furthermore, we also explored whether aging may affect the ability to use contextual information to predict the outcome of observed actions ahead of realization (Amoruso et al., 2016, 2020). Based on the aging-related effects on predictive coding (Chan et al., 2021; Gilbert and Moran, 2016; Hsu et al., 2021; Trapp et al., 2022), we expected that any decline in the ability to process sensory information about action kinematics could be compensated by greater reliance on contextual information. Furthermore, the use of contextual information should correlate with visual imagery and level of action identification. By examining behavioral parameters such as sensitivity, criterion, and reaction time, we seek to identify any decline in behavioral performance during lifespan, thereby providing further insights into the influence of aging on action prediction.

## 2 Materials and methods

### 2.1 Study design

This study was developed with a single-centre observational cross-sectional design. The study procedures were approved by the local Ethics Committee (CERA, Unige) and were carried out in accordance with the Declaration of Helsinki (World Medical Association, 2013). Written informed consent to research and to publication of results was obtained from all participants before to be included in the study project.

### 2.2 Participants

All participants were recruited at the University of Genoa between July 2022 and November 2023 from within speech therapy and physical therapy degree programs, students’ parents or relatives, and through direct contacts of members of the study research team. Healthy individuals, aged between 18 and 30 (young adulthood), and 46–80 years old (middle and late adulthood), for younger and older group respectively, were included in this study. Inclusion criteria required the absence of major neurological and psychiatric disorders and of any musculoskeletal or orthopaedic condition that could restrain the participants’ upper-limb mobility. The participants’ enrollment was inclusive of all individuals, irrespective to their gender or ethnicity. Thirty younger (13 females, mean age 22.90 ± 2.87 years, median age 22 years) and 30 older healthy participants (15 females, mean age 60.20 ± 8.18 years, median age 60.5 years) took part in the study. All the participants were tested with the Edinburgh Handedness Inventory (EHI) questionnaire (Oldfield, 1971) and reported normal or corrected-to-normal vision. No information about the racial distribution of the sample were recorded. Demographic characteristics and behavioral outcome measures of participants are reported in Table 1. Screening for age-related neurocognitive decline was conducted using the Montreal Cognitive Assessment (MoCA) (Nasreddine et al., 2005) among older participants. Prior to participation, all participants were naïve to the study aim and hypotheses and were informed about the study purposes at the end of the experimental procedures.

**Table 1:** Participants’ demographics and behavioral characteristics.

	Young (*n* = 29)	Older (*n* = 28)	Statistics
Gender M:F *n* (%)	13:16 (45, 55)	13:15 (46, 54)	c^2^ = 0.01, *p* = 0.90
Age (y) mean (±SD)	22.97 (±2.90)	60.64 (±8.18)	t_58_ = −23.34, *p* < 0.001
Education (y) mean (±SD)	15.28 (±2.14)	13.82 (±3.34)	t_58_ = 1.98, *p* = 0.05
EHI R:L n (%)	24:5 (83, 17)	27:1 (96, 4)	c^2^ = 2.83, *p* = 0.09
MoCA (score) mean (±SD)		27.18 (±1.31)	
KVIQ-k mean (±SD)	30.34 (±8.35)	25.46 (±11.46)	t_58_ = 1.84, *p* = 0.07
KVIQ-v mean (±SD)	34.79 (±11.10)	40.46 (±11.12)	t_58_ = −1.93, *p* = 0.06
BIF [mean (±SD)]	57.66 (±20.33)	65.29 (±19.99)	t_58_ = −1.43, *p* = 0.16

M, male; F, female; n, numerosity; y, years; SD, standard deviation; EHI Edinburgh Handedness Inventory; R, right; L, left; BIF, Behavioral Identification Form; KVIQ-k/v, visual/kinesthetic subscale of the Kinesthetic and Visual Imaginary Questionnaire. Statistically significant difference: *p* < 0.05.

### 2.3 Stimuli

Experimental videos of this project were validated and used in a previous study (Bianco et al., 2024). Briefly, the videos showed a female (aged 36 years) or a male (aged 43 years) actor performing reach-to-grasp movements toward a krapfen (big object), placed on a plate, or one of two cream puffs (small objects) placed in a transparent bowl, with his/her right hand. The right upper limb was the only actor’s body parts visible in the videos. Two alternative actions were recorded, reaching and grasping the food with the intention to eat, or reaching and grasping the food container with the intention to move. Notably, the patterns of reach-to-grasp movements varied depending on the size of the food item and the design of the container. According to the object, each action was associated with specific kinematics and type of grasping: the action to eat required a whole-hand grip for grasping the krapfen, and a precision grip for grasping the cream puff; the action to move required a precision grip of the krapfen plate and a whole-hand grip of the puff cream bowl. Also, two scenarios were created: a set table indicating an intent to eat, and a cleared table indicating an intent to move. The set table included a teacup full of tea, a closed teapot, an empty saucer, and a tray, suggesting a meal yet to be consumed. The cleared table included an empty teacup, an open empty teapot, a used teabag on the saucer, and a tray, suggesting a meal already consumed. Accordingly, the intentions suggested by the two different contextual scenarios could be congruent or incongruent with the intentions behind the actual action kinematics. As an example, an action congruent with the context was grasping a food with the intention to eat in a set table, whereas an incongruent action was grasping a food with the intention to eat in a cleared table. It is important to note that contextual congruence was relevant only in relation to the kinematics of the movement, not in terms of its inherent plausibility. In the congruent condition, the contextual constraints matched with the observed kinematics. In the incongruent condition, the contextual information interfered with the perception of the observed kinematics by cueing to the opposite action.

To summarize, there were six distinguishable variables in the videos, and each one could be expressed in two different ways. The combination of the following factors produced 32 distinct videos: object (cream puff or krapfen), context (congruent or incongruent), video interruption point (early or late), type of grasping (precision or whole-hand), intention of the action (eat or move), and gender of the actor performing the movement (male or female). Participants observed each of the 32 videos four times in a random order, for a total of 256 trials.

### 2.4 Procedure

The whole experimental session lasted approximately 60 min. In this session, participants performed the action prediction task (about 15 min.) and demographical characteristics, imaginative ability and individual differences in action identification were measured.

#### 2.4.1 Action prediction task

**Figure 1:**
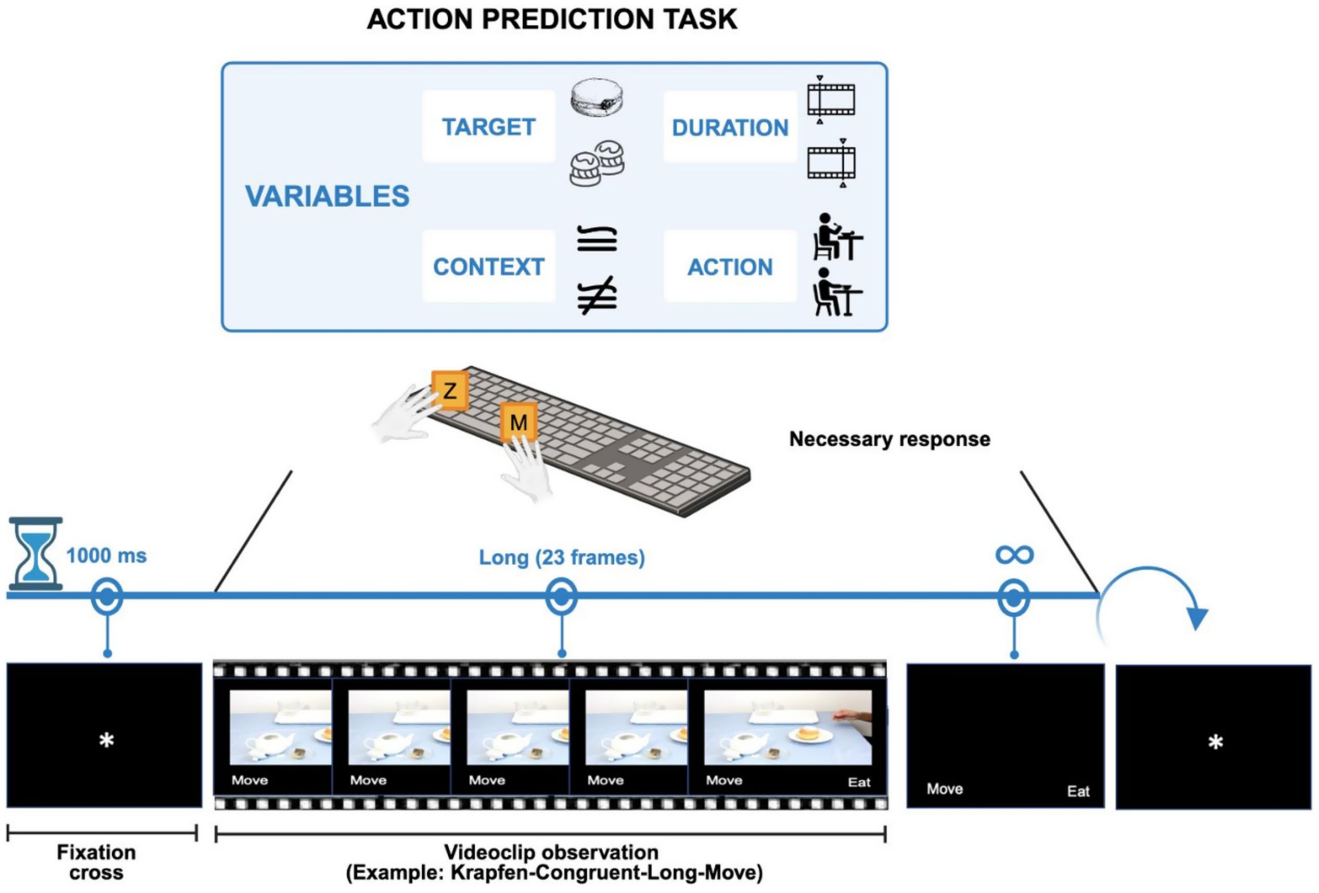
Schematic representation of the action prediction task. The figure shows the experimental task. The time sequence of the experiment is depicted on the blue line with the hourglass. The black screen with the fixation cross, lasting 1,000 ms, indicates the start of the task. The videoclip illustrates one of the 256 trials. The icons in the box above depict the combinable variables of the videos: object (krapfen or cream puffs), interruption point (early with 13 frames or late with 23 frames), action (to eat or move) and context (congruent or incongruent). The black screen with the two descriptors “move” and “eat” shows the two possible answers. “z” or “m” are the keys to give the answer. A response is required without any time limit (infinite symbol). The round arrow and the last black screen with the fixation cross indicate the continuation of the task.

The experimental paradigm is depicted in Figure 1. Participants were seated in a chair in a quiet room approximately 1 meter from the screen. Action prediction ability was tested using a two-Alternative Forced Choice (2AFC) task (Amoruso et al., 2016; Amoruso and Urgesi, 2016; Bianco et al., 2024). Participants were instructed to carefully read the instructions on the screen, to observe the videos and predict which one of the two possible intentions (i.e., to eat or to move) was behind the actor’s movements.

At the beginning of the task, simultaneously with the appearance of the video, a frame with the verbal descriptors of the alternative action intentions (i.e., “move” and “eat”; in Italian, “mangia” e “sposta”) written in white on a black background was presented. This frame remained on the screen until a response was recorded. The verbal descriptors were displayed on the left and right of the screen, and the location was counterbalanced among subjects.

Using a temporal occlusion paradigm, the videos ended before the actor reached the objects. Specifically, the video presentation could be interrupted at an early (frame 13) or late (frame 23) action phase. The time at which to set the two cutoff points (early and late) was chosen based on the pilot part of a previous study by Bianco and colleagues (Bianco et al., 2024 in Supplementary material). Several observed video interruption points were compared in that study, and the two most significant ones were selected and employed in their primary investigation. In the early interruption, the actor was at the very beginning of the reaching movement, providing ambiguous, still available, kinematic information, whereas in the late interruption, the actor’s hand was nearly approaching the object, making the pre-shaping of the hand configuration more informative about the two action intentions. A previously reported kinematic analysis of the action videos used in this study [see Supplementary materials in Bianco et al., (2024)] showed higher thumb opponency of the index and little fingers and higher height of the wrist during whole-hand than precision grips. Although these differences were progressively enhanced with action unfolding, kinematic parameters differentiated the two types of grips already at the early interruption point (i.e., frame 13). Furthermore, electromyography recording during execution of the same actions (Bianco et al., 2024) revealed that, already in the reaching phase, the first dorsal interosseous muscle (which is involved in the movement of the index finger) was more engaged during precision (i.e., eating the cream-puff and moving the plate containing the krapfen) than whole-hand grips (i.e., moving the bowl containing the cream-puffs and eating the krapfen). Conversely, the abductor digiti minimi and the extensor carpi radialis, involved, respectively, in movements of the little finger and of the wrist, were more activated for actions involving a whole-hand grip than for actions involving a precision grip. Notably, duration of the videos was kept constant at 800 ms across interruption points by lengthening the presentation of the first frame (showing a still hand) in the early interruption point videos. This way, any difference between the two types of videos was related to the amount of kinematic information and not to video duration and availability of contextual information. Before starting the task, all participants were informed that the videos would be interrupted before action completion and were encouraged to predict the action intention based on the available information in the videos (e.g., kinematics and contextual information). Also, they were informed that some items in the scene (i.e., a teapot, a teacup, and a tea bag) were presented in different scenarios: a set or a cleared table. Conversely, the association between contextual cues and action intentions was not explicitly encouraged.

Each trial started with a 1-s fixation point (i.e., a white cross), followed by the presentation of the action videos (for 800 ms) and of a black screen with the two response alternatives until response. To provide their responses, participants had to press the computer keys “z” (for left choices) or “m” (for right choices) with their index finger. Following participant’s response, a white cross appeared for 1 s.

The total number of 256 trials were randomly presented in four blocks of 64 trials each. Throughout the experiment, participants did not receive any feedback on their performance. Stimuli were presented using E-Prime 3.0 software (Psychology Software Tools, Pittsburgh, PA) on a 24-inch LCD monitor (resolution, 1920 × 1,080 pixels; refresh frequency, 120 Hz).

#### 2.4.2 Other behavioral measures

Before the action prediction task, imagery ability was investigated via the Kinesthetic and Visual Imaginative Questionnaire (KVIQ) (Malouin et al., 2007), a five-point ordinal scale in which participants should mentally represent ten simple bodily movements. Firstly, the clarity of the visual representations was rated using a Likert-like scale which ranges in score from one, indicating “no image,” to five, suggesting “image as clear as seeing”; then, the intensity of the kinesthetic sensations was rated using a similar scale ranging from one, signifying “no sensation,” to five, meaning “as intense as executing the action.”

Finally, the Behavioral Identification Form (BIF) (Vallacher, 1989) was used to assess individual differences in characteristic level of action identification. The BIF questionnaire is composed by 25 items, scored 0 or 1, in which different activities are proposed. Participants choose between two answers that describe two different styles of an action: one more concrete, low-level, and detailed (0 point), the other more abstract, high-level, related to the consequences of the action (1 point). According to the Action Identification Theory (Vallacher and Wegner, 1987, 2012; Wegner et al., 1986) lower scores are associated to poorer and more hesitant action performance.

### 2.5 Outcome measures

#### 2.5.1 Action prediction task

In the action prediction task, we calculated the percentage of correct responses for each action and the means of Reaction Time (RT) of correct answers, reported in milliseconds (ms). Data processing was executed using E-Prime 3.0 software (Psychology Software Tools, Pittsburgh, PA). Trials with RTs < 250 ms or > 5.000 ms were considered, respectively, accidental button presses or missed responses and were removed from the analysis. Performance data were treated according to the Signal Detection Theory (SDT) (Macmillan and Kaplan, 1985; Stanislaw and Todorov, 1999) by calculating d prime (*d*′) and criterion values. The *d*′ value represents a bias-corrected measure of sensitivity in discriminating between 2 categories and higher values of *d*′ are indicative of a greater sensitivity. In the *d*′ analysis, “eat” identified as “eat” ‘were considered as “hits,” videos with “move” identified as “eat” were considered as “false alarms.” The *d*′ values were calculated by transforming the response proportion to *z*-scores, and then subtracting the z-score that corresponds to the false-alarm rate from the *z*-score that corresponds to the hit rate (Stanislaw and Todorov, 1999). Furthermore, the measure of response criterion (c) reflects the existence of a bias in providing a specific response with negative values pointing to a tendency toward reporting the signal (i.e., to eat) and positive values a tendency toward reporting the absence of the signal (i.e., to move); values close to 0 suggest no bias. The c values were calculated by averaging the z-score corresponding to the hit rate and the z-score corresponding to the false-alarm rate, and then multiplying the result by −1 (Stanislaw and Todorov, 1999).

#### 2.5.2 Imagery ability and level of action identification

The KVIQ was scored by summing the scores at each of the 10 items of the two subscales to calculate the visual (KVIQ-v) and kinesthetic (KVIQ-k) sub-scores, each ranging from 10 to 50; the greater the number, the more proficient the individual motor imagery is.

The total BIF score was determined by counting how many high-level alternatives are chosen; higher scores suggest a greater inclination to understand other action in terms of its consequences and implications, rather than in terms of its details and mechanics.

### 2.6 Data analysis

The sample size was estimated using the G*Power 0.13 software (Faul et al., 2007), with the “as in SPSS” option. A sample of 60 participants was considered adequate to achieve a power of 80% (1 − *β* = 0.80; *α* = 0.05), for testing 2-way between-within interactions in our mixed repeated-measure analysis of variance (RM-ANOVA) design (numerator df = 1) with an estimated large effect size (fU = 0.4) and a potential drop-out rate of 10%. The effect size was estimated based on the partial *η*-squared (η^2^_p_) of the contextual modulation effect reported in previous studies (main effect of contextual probability in Amoruso et al., 2019: η^2^_p_ = 0.1; interaction between interruption time and contextual congruency for the behavioral data in Amoruso et al., 2018: η^2^_p_ = 0.13; interaction between interruption time and contextual congruency in the pilot behavioral experiment in Bianco et al., 2024; average η^2^_p_ = 0.14). This led to a minimal sample size of 54 participants, which we increased to 60 to compensate for any drop-out.

Demographic data are reported as frequencies and percentages (%) for dichotomous variables, or as mean values and Standard Deviation (SD) for continuous variables. Group comparisons for gender (M: F) were conducted using the chi-square test, while independent-samples t-tests (two-tailed) were used to compare age, years of education, and behavioral measures (KVIQ and BIF).

Performance at the action prediction task, measured by *d*′, c and RTs, was analyzed with a 2 × 2 × 2 mixed RM-ANOVA, with GROUP (young and older adults) as between-subject factor and Context (congruent or incongruent) and Interruption (early or late) as within-subject variables. Post-hoc analysis was performed using the Holm-Bonferroni correction method for multiple comparisons and data were reported as mean ± Standard Error Mean (SEM).

Prior to conducting statistical analyses, data were screened for assumptions of normality and the presence of outliers. Normality of the distributions was assessed using Shapiro–Wilk test. Outliers were identified based on the ±2 SD criterion from the mean for each variable and were excluded from specific analyses as appropriate. Estimates of the effect size were reported using the η^2^_p_ for main effects and interactions and Cohen’s d for pairwise comparisons.

To explore possible relationship between action prediction performance and behavioral measures, context effect indices were computed by subtracting performance scores for incongruent-context trials from congruent-context trials (for *d*′, c, and RTs). Pearson correlations were then calculated between the context effect indexes for each performance measure at the two interruption points and the KVIQ-v, KVIQ-k and BIF scores across groups. Specifically, partial correlations were computed controlling for the continuous variable age, to statistically remove its potential confounding effect on these relationships. A Bonferroni correction procedure was used to control for multiple correlation testing for each performance measure (i.e., 3 questionnaires x 2 interruption points). All inferential tests of significance were based upon an *α* level of 0.05.

Computerized tasks were run using E-Prime 3.0 software (Psychology Software Tools, Pittsburgh, PA). Statistical analyses were performed using JASP version 0.19.0 (JASP Team, 2024). All deidentified data are available on the Open Science Framework repository at https://osf.io/umny6/. The study and analytic plan were not preregistered.

## 3 Results

Outlier detection resulted in the exclusion of three participants (two older adults and one younger adult). Consequently, the analyses were conducted on 29 younger participants (16 females, mean age 22.97 ± 2.90 years, median age 22 years) and 28 older healthy participants (15 females, mean age 60.64 ± 8.18 years, median age 61 years).

The two age groups were comparable for gender distribution and education; although the older group tended to have lower education levels, this difference was not statistically significant. Both groups exhibited similar levels of action identification and intensity of kinesthetic sensations during motor imagery; however younger participants reported lower vividness of visual representation during motor imagery (Table 1).

### 3.1 Action prediction outcome measures

#### 3.1.1 Sensitivity *d*′

**Figure 2:**
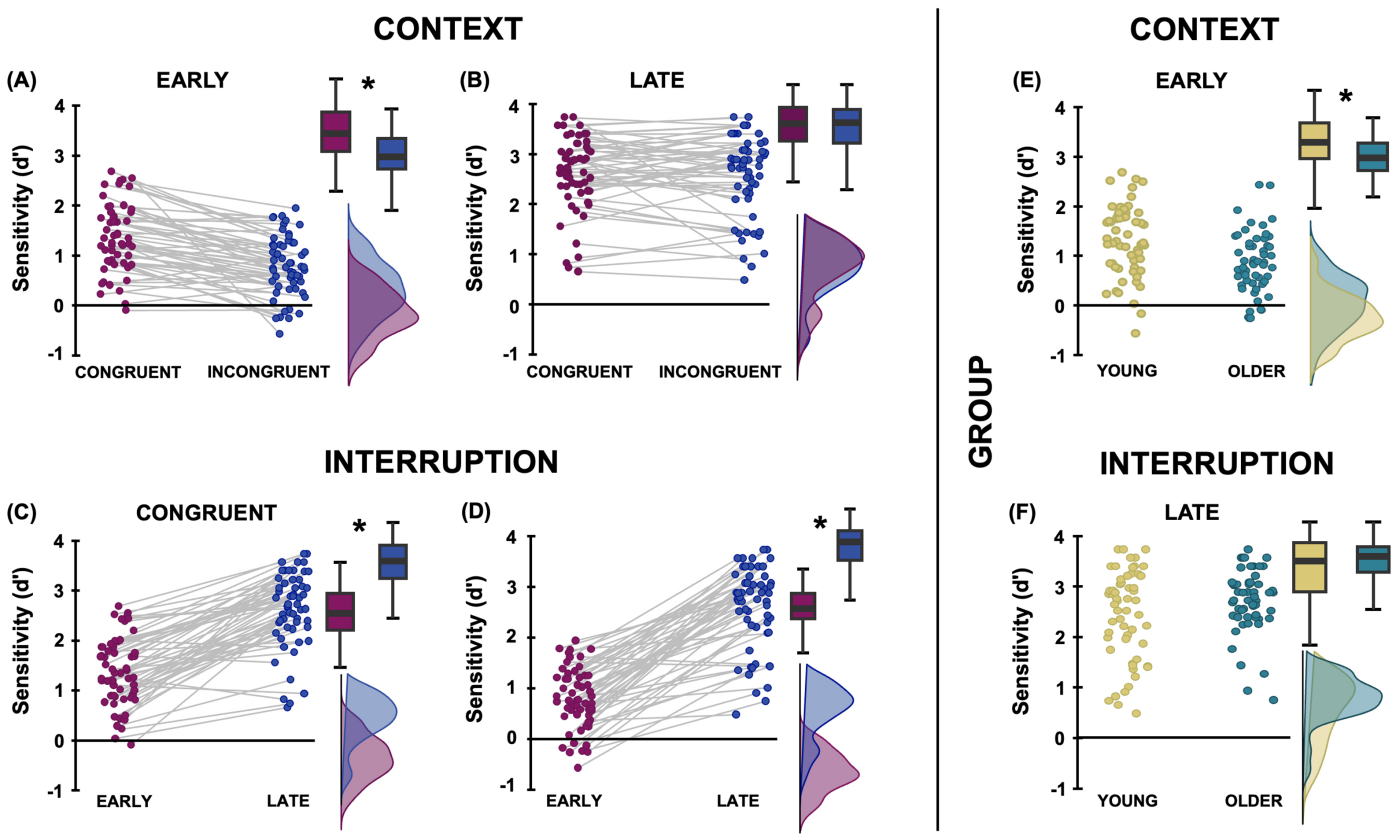
Graphical representation of the results on sensitivity (*d*′). Panels **(A,B)** illustrate the raincloud plots of the significant 2-way interaction between Context and Interruption, showing the effect of Context at Early and Late Interruption level, collapsing across the two groups. Panels **(C,D)** show the raincloud plots of the same significant 2-way interaction between Context and Interruption, considering the effects of Interruption at Congruent and Incongruent Context level. Panels **(E,F)** show the raincloud plots of the significant 2-way interaction between Group and Interruption, collapsing context types, providing between-group (young-yellow; older-petrol) comparisons. Boxplots and overlaid density distributions illustrate the central tendency and distribution of sensitivity scores across conditions. Asterisks denote significant post-hoc comparisons.

The analysis of sensitivity (Figure 2A) revealed a significant intercept term (F_1,55_ = 766.08, *p* < 0.001, η^2^_p_ = 0.922), suggesting that participants’ performance was overall greater than chance (i.e., *d*′ > 0). The analysis also yielded significant main effects of Context (F_1,55_ = 23.05, *p* < 0.001, η^2^_p_ = 0.30) and Interruption (F_1,55_ = 326.85, *p* < 0.001, η^2^_p_ = 0.86), which were better qualified by their significant interaction (F_1,55_ = 31.49, p < 0.001, η^2^_p_ = 0.36; Figure 2B). Holm-Bonferroni post-hoc analysis showed that sensitivity was higher for predicting actions embedded in congruent than incongruent scenarios at early (1.36 ± 0.64 vs. 0.79 ± 0.57; *p* < 0.001; Cohen’s d = 0.82), but not at late (2.62 ± 0.73 vs. 2.57 ± 0.81; *p* = 0.592; Cohen’s d = 0.06) interruptions. Furthermore, participants were more sensitive to action deployment in both congruent and incongruent contexts when the videos were interrupted in the late than early interruption point (both *p* < 0.001). The main effect of Group was not significant (F_1,55_ = 0.27, *p* = 0.608, η^2^_p_ = 0.005). In addition, the interactions between Group and Context and between Group, Context and Interruption were not significant (all F_1,55_ < 1), suggesting that the effect of contextual modulation was similar across the two age groups. Notably, however, the interaction between Group and Interruption was significant (F_1,55_ = 17.83, *p* < 0.001, η^2^_p_ = 0.24; Figure 2C). Holm-Bonferroni post-hoc analysis showed that older adults had lower sensitivity compared to the young ones when actions were interrupted early (0.86 ± 0.56 vs. 1.29 ± 0.65; *p* = 0.023; Cohen’s d = 0.62) but not later (2.74 ± 0.64 vs. 2.45 ± 0.90; *p* = 0.096; Cohen’s d = −0.40). Moreover, both groups improved with later interruption (all p < 0.001; Cohen’s d > −1.66).

#### 3.1.2 Criterion

**Figure 3:**
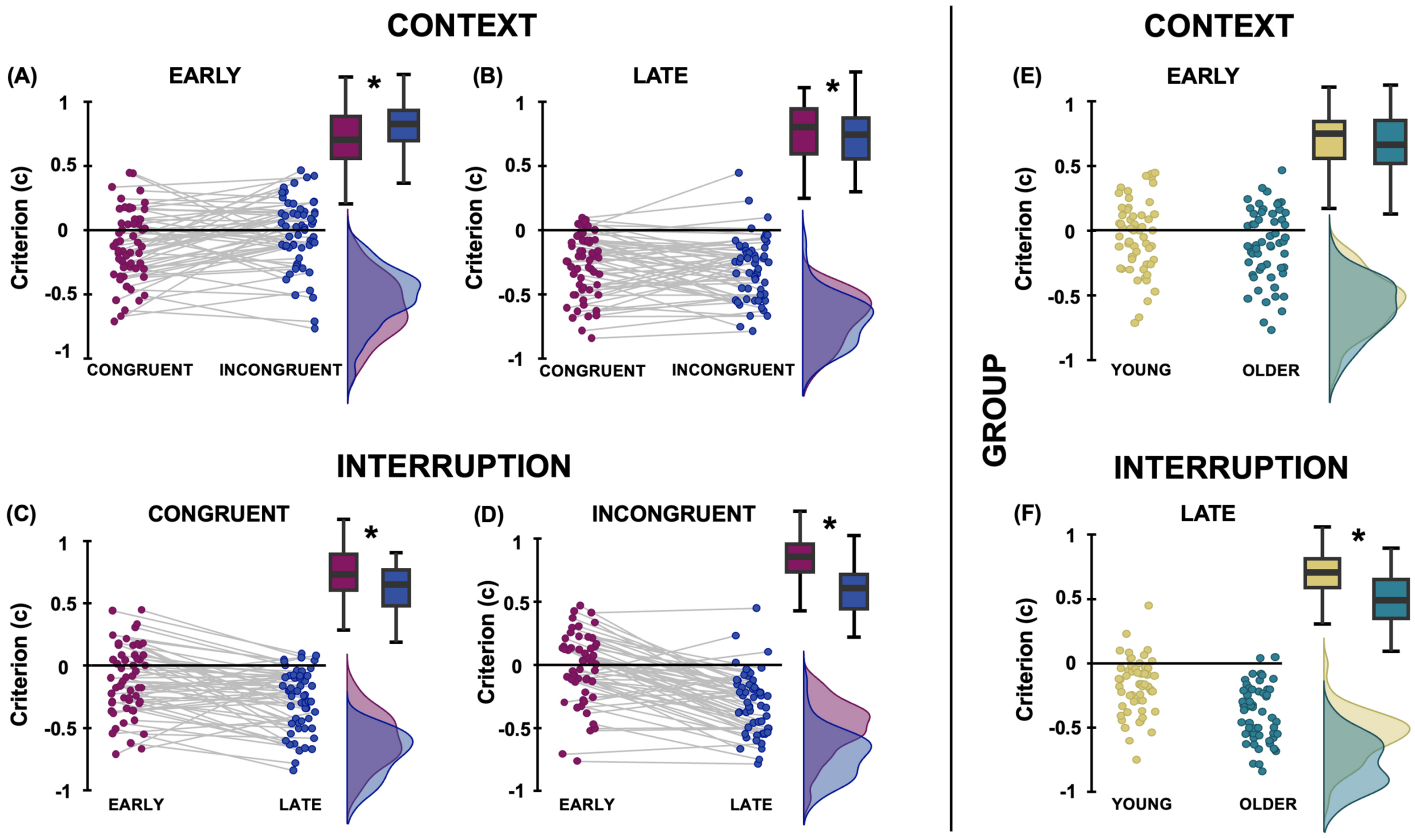
Graphical representation of the results on criterion (c). Panels **(A,B)** illustrate the raincloud plots of the significant 2-way interaction between Context and Interruption, showing the effect of Context at Early and Late Interruption level, collapsing across the two groups. Panels **(C,D)** show the raincloud plots of the same significant 2-way interaction between Context and Interruption, considering the effects of Interruption at Congruent and Incongruent Context level. Panels **(E,F)** show the raincloud plots of the significant 2-way interaction between Group and Interruption, collapsing context types, providing between-group (young-yellow; older-petrol) comparisons. Boxplots and overlaid density distributions illustrate the central tendency and distribution of sensitivity scores across conditions. Asterisks denote significant post-hoc comparisons.

The analysis on Criterion (c; Figure 3A) showed a significant Intercept effect (F_1,55_ = 51.16, *p* < 0.001, η^2^_p_ = 0.482), revealing an overall negative c, which suggests a bias to report an eating action across conditions and groups in our action prediction task. Context had no significant main effect (F_1,55_ = 2.17, *p* = 0.146, η^2^_p_ = 0.04), but it significantly interacted with Interruption (F_1,55_ = 5.41, *p* = 0.024, η^2^_p_ = 0.09; Figure 3B). Post-hoc test revealed that, collapsing across age groups, c was more negative at late than early interruptions for both congruent (−0.28 ± 0.22 vs. −0.12 ± 0.28; *p* < 0.001; Cohen’s d = 0.62) and incongruent contexts (−0.03 ± 0.21 vs. −0.02 ± 0.27; p < 0.001; Cohen’s d = 1.13). Furthermore, c was more negative for predicting actions embedded in congruent than incongruent contexts only at the early (*p* = 0.015; Cohen’s d = −0.39), but not at the late (*p* = 0.375; Cohen’s d = 0.13) interruption.

The mixed RM-ANOVA also showed that the main effects of Group (F_1,55_ = 8.20, *p* = 0.006, η^2^_p_ = 0.13) and Interruption (F_1,58_ = 102.16, *p* < 0.001, η^2^_p_ = 0.637) were significant and were further qualified by their significant interaction (F_1,58_ = 14.91, *p* < 0.001, η^2^_p_ = 0.21; Figure 3C). Holm-Bonferroni post-hoc analysis showed that the young-adult group had more negative c values than the older-adult group for late-interrupted actions (−0.41 ± 0.22 vs. −0.18 ± 0.21; p < 0.001; Cohen’s d = 0.93), but not for early ones (−0.11 ± 0.28 vs. -0.04 ± 0.27; *p* = 0.43; Cohen’s d = 0.26); both groups had a more negative c value for the late than the early interruption (all *p* < 0.001; Cohen’s d > 0.54). All other effects were not significant (all F_1,55_ < 1).

#### 3.1.3 Reaction time

**Figure 4:**
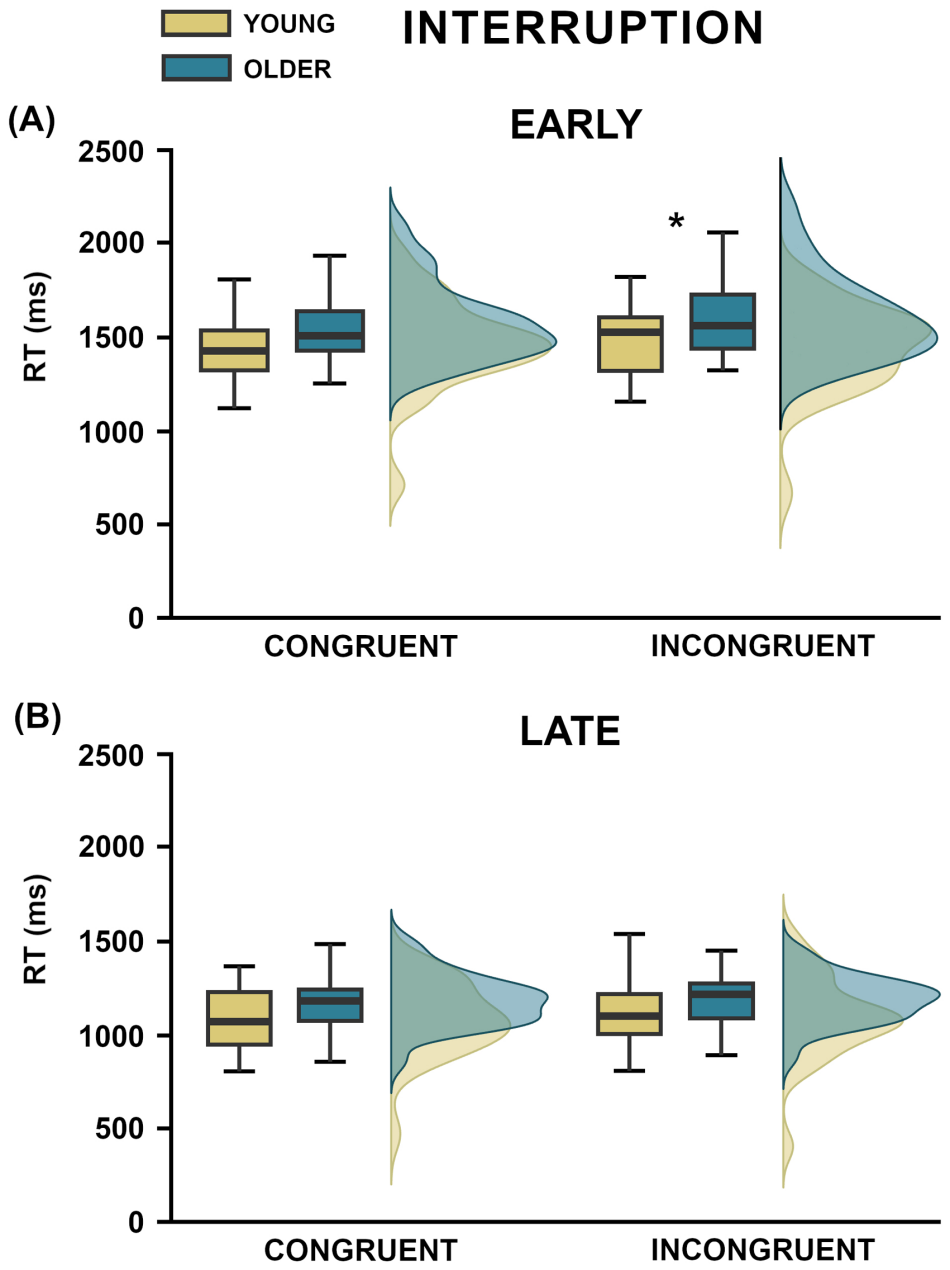
Graphical representation of the results on reaction times (RTs, ms). Panels **(A,B)** show the boxplots and overlaid density distributions of the significant 3-way interaction between Group, Context and Interruption, comparing congruent and incongruent contexts at early **(A)** and late **(B)** interruption points within the young (yellow) and older (petrol) groups. Asterisks denote significant post-hoc comparisons.

The analysis of RTs yielded significant main effects of Context (F_1,55_ = 24.94, *p* < 0.001, η^2^_p_ = 0.31) and Interruption (F_1,55_ = 771.26, *p* < 0.001, η^2^_p_ = 0.93), pointing to overall faster responses for congruent compared to incongruent contexts, and to late compared to early interrupted actions. In addition, the main effect of Group was significant (F_1,55_ = 5.19, *p* = 0.027, η^2^_p_ = 0.09), further qualified by a marginally significant three-way interaction Group x Context x Interruption (F_1,55_ = 4.02, *p* = 0.049, η^2^*_p_* = 0.07; Figure 4), suggested that the response speed of young and older participants was differently affected by context at the two interruption points. Holm-Bonferroni post-hoc analysis showed that the between-group differences did not reach significance in any condition (all *p* > 0.29, Cohen’s d < −0.49), except in the incongruent context at early interruption in which older group recorded slower responses compared to younger individuals (1609.17 ± 42.08 ms vs. 1447.99 ± 43.45 ms; *p* = 0.038, Cohen’s d = −0.8). Generally, both groups were faster at predicting late- than early-interrupted actions (all p < 0.001). However, while the young-adults’ speed of response was not affected by contextual congruency at either early (*p* = 0816) or late (*p* = 0.081) interruption point, the older-adult group (Figure 4C) was faster for congruent than incongruent contexts at early (1555.35 ± 38.22 ms vs. 1609.17 ± 42.08 ms; *p* = 0.001, Cohen’s d = −0.27), but not at late (1155.02 ± 26.44 ms vs. 1182.71 ± 23.46 ms; *p* = 0.29, Cohen’s d = −0.14) interruption point. All other ANOVA effects were non-significant (all F_1,55_ < 3.28, *p* > 0.07, η^2^_p_ < 0.06).

#### 3.1.4 Correlations

Correlational analyses were conducted to examine the relationships between the context effect indices for *d*′, c, and RTs and the behavioral measures (KVIQ-v, KVIQ-k, and BIF), both across the entire sample and within each age group separately. These analyses did not reveal any statistically significant associations after correction for multiple comparisons (all *p* > 0.3).

## 4 Discussion

### 4.1 Brief general summary

The aim of this study was to better understand how physiological aging shapes action prediction abilities. Specifically, we investigated whether kinematic and contextual information differently influence intention understanding in young and older individuals, highlighting potential age-related differences in these influences. Participants were instructed to watch videos and predict the outcome of an action before it occurred, using a two-alternative forced-choice task with temporal occlusion (Amoruso et al., 2016, 2018; Betti et al., 2022). The tasks involved observing videos of familiar object-oriented actions commonly experienced in everyday life (i.e., breakfast scenario). In these videos, kinematics of the observed movements was manipulated by showing actions directed towards a big or a small object (i.e., krapfen or cream puff) with the intent to eat or to move it. Actions were embedded in different contextual scenarios, which pointed to an intention that was either congruent or incongruent with that suggested by action kinematics. Furthermore, action deployment was interrupted at an early or a late unfolding phase, thus providing more or less ambiguous kinematic information.

Overall, we found that contextual congruency impacted the action prediction performance of both groups in terms of sensitivity and response speed, especially when actions were interrupted at early phases ahead of realization and, thus, when the ambiguity of kinematic information challenged participants’ prediction performance. However, age-related differences emerged, since older participants were less sensitive than the young ones to the early kinematics of action unfolding, showing reduced performance for early-interrupted videos. This reduced ability to read out the initial phases of action unfolding co-occurred with a greater modulation of their response speed by contextual congruency, suggesting a compensatory mechanism related to aging.

Furthermore, while young adults showed a general bias to report an eating rather than a moving action, older adults were less affected by this response bias, particularly when more kinematic information was available in late-interrupted actions. In sum, older adults demonstrated aging-related alterations in their sensitivity to early kinematic information in the prediction of action unfolding ahead of realization, but they benefited more from contextual information. Contrary to our expectations, the contextual modulation effects did not correlate with measures of level of action identification or imagery abilities.

### 4.2 Contextual modulation of action prediction ability

Our study found that sensitivity in discriminating between two very similar reaching-to-grasp actions performed with a different intent (i.e., to eat or to move) was good (*d*′ > 0) in both groups of participants, confirming that they were able to read out early kinematic information to predict action unfolding ahead of realization. Performance was better at the late than the early interruption point, suggesting that action prediction ability depends on the type and amount of available kinesthetic information (Amoruso et al., 2016; Betti et al., 2021; Bianco et al., 2024; Stapel et al., 2015). Performance was good also at the early interruption point, being above chance even when kinematics was embedded in incongruent contexts. This aligns with previous evidence that even limited kinematic information can be used by observers to discriminate between alternative action intentions (Cavallo et al., 2016). Consistent with our examination of how aging interacts with contextual information processing, we noted that contextual information heavily modulated participants’ prediction of action outcomes, refining and speeding up performance when context was congruent with kinematics and blurring and slowing down it for incongruent contexts. Importantly, contextual information modulated performance not only at the early interruption, when kinematics was very ambiguous, but also at the late interruption, when action unfolding provided more reliable information about the action intention and the observers were very good at predicting its outcome (i.e., *d*′ > 2). This suggests that contextual information serves action prediction not only in conditions of perceptual ambiguity (Cavallo et al., 2013; Cretu et al., 2019; Koul et al., 2019), but it is continuously integrated with the unfolding kinematics in order to represent the ultimate action intention and ensure the most accurate and fast action understanding (Alhasan et al., 2025; Bianco et al., 2024).

The response bias that all participants, particularly the young ones, exhibited as a tendency to report more an eating than a moving action highlights that observers enter action prediction with their own expectations based on prior experience and knowledge. Indeed, numerous studies have demonstrated that predictive processes are context-sensitive and influenced by prior experience of the observed action (Amoruso et al., 2014; Iacoboni et al., 2005; Kilner et al., 2007; Paolini et al., 2023). Since we used food objects, it is likely that perceptual predictions were biased toward the primary function (i.e., to eat) of this object (Alhasan et al., 2025). This is in keeping with findings showing that the affordances (i.e., the relational action possibilities that the environment offers to an organism, relative to its capabilities) defined by the target object may influence both perceptual predictions (Alhasan et al., 2025; McDonough et al., 2020) and motor responses (Ruggiero and Catmur, 2018) during action observation. However, since we miss a non-food object as a control, we cannot exclude that the bias toward an eating than a placing action may reflect the different valence of the two actions (e.g., eating is likely more positive than clearing the table) or a general tendency to prioritize actions that are more salient from an evolutionary point of view. Indeed, studies in monkey (Fogassi et al., 2005) have found more neurons in the inferior parietal lobe that respond during the execution and observation of grasping actions when embedded in an eating, compared to a placing motor chain; interestingly, these neurons responded more when the target object of a placing action was a piece of food compared to a solid object. Nevertheless, we found that the bias for predicting an eating action was stronger at the later compared to the early interruption point, thus it increased when more kinematics was available from the video. Furthermore, at the early interruption point, it was stronger in the young-adult than in the older-adult group, thus it was stronger in the group who was more sensitive to kinematic information during early action prediction. This keeps with the notion that actions are perceived as context-embedded, with object affordances, actors, kinematics, time, location, and the relationships amongst them ‘gluing together’ into a unifying scene (Alhasan et al., 2025; Amoruso et al., 2018; Ibañez and Manes, 2012). Our findings show that aging and experience may shape how these elements are integrated during the prediction of others’ behaviors.

### 4.3 Age-related differences in sensitivity to action kinematics

When comparing behavioral performance between groups, our results showed that aging significantly impacts the ability to accurately predict the outcome of an unfolding action. Specifically, older adults were less sensitive compared to young participants in predicting the action intention from limited kinematic information, regardless of the type of context. Action prediction performance of the two age groups was comparable when more kinematic information was provided (i.e., late-interrupted videos). However, older adults were less sensitive than young adults when action unfolding was interrupted early and they received limited kinematic information (i.e., early-interrupted videos).

These findings are consistent with previous behavioral and neurophysiological studies investigating changes in action prediction processes over the lifespan (Diersch et al., 2012, 2013; Sacheli et al., 2023). Diersch et al. (2012, 2013) demonstrated that older adults predicted observed action sequences less precisely than younger adults, even when they were familiar with the actions. Moreover, neurophysiological results revealed that, during the observation of familiar actions, older adults recruited visual regions, the hippocampus, and the caudate more than younger adults. This may be due to older adults relying predominantly on the visual dynamics of the observed actions during the occlusion period instead of effectively exploiting the sensorimotor matching properties of the AON (Grafton, 2009). More recently, Sacheli et al. (2023) investigated whether visuo-motor mechanisms involved in action prediction degrade with aging. Specifically, they studied age-related decline in the ability to anticipate the unfolding of overlearned simple movements (i.e., grasping or pointing to a simple cubed-shaped object placed in the virtual peri-personal space of the observer) comparing the performance with a control color-discrimination task. Results showed a significant difference between younger and older participants, with lower performance in the older group. However, no significant group-by-task interaction was found, suggesting that the reduced performance shown by the older participants was not specific to a decay in action prediction abilities, but it rather reflected a broad generalized decline in performance. Conversely, neuroimaging results showed age-related differences specific to action prediction. Aging was associated with under-recruitment of visuo-motor mirror mechanisms in the AON when predicting the conclusion of simple gestures, compared to the control task (Diersch et al., 2013, 2016).

Taken together, our results, alongside previous research, suggest that aging primarily affects the ability to use early kinematic information to predict action unfolding. While we cannot rule out that this reduced sensitivity reflects a general visual discrimination impairment, as shown by Sacheli et al. (2023), older adults showed comparable sensitivity to kinematic information conveyed by late stages of the actions, despite the fact the video duration was kept constant. Furthermore, older adults were able to visually process contextual objects and to use this information to predict actions. Indeed, the congruence between the action kinematics and the contextual information affected older-adults’ sensitivity comparably to that of the young-adults. If their performance pattern reflected general visual defects, older-adults should be less sensitive to the information provided by the contextual objects and show weaker contextual modulation of performance. In fact, their speed of response was more affected by contextual information compared to that of young individuals at the early interruption point. This may indicate that they could extract visual information from the context to guide the prediction of the upcoming actions. Thus, the adults’ weakness in processing ambiguous action kinematics did not seemingly extend to the visual processing of contextual objects.

### 4.4 Age-related differences in context-based action prediction

An important finding of this study is that aging affected the use of contextual information during the action prediction task. While contextual modulation was comparable between the two age groups in terms of sensitivity, the (in)congruence of contextual cues with action kinematics affected response speed of older adults for both early- and late-interrupted actions, while it was reliable only at the late interruption point for young adults. Thus, compared to young adults, older adults were less sensitive to early kinematic information in predicting action unfolding and tended to use in more immediate ways contextual cues to resolve the ambiguity of perceptual information. Within a predictive coding account of action understanding (Kilner et al., 2007), this suggests that aging may lead to a greater weighting of contextual expectations compared to incoming sensory information. This could reflect either a compensation for blurred sensory inputs with the aim of saving performance (Davis et al., 2008) or the consequence of a refinement across the lifespan of the internal modelling of the external world to optimize predictions (Moran et al., 2014). The fact that the greater use of contextual information in older participants for the early-interrupted actions was accompanied by a reduced sensitivity across context conditions would favor a compensation explanation.

Our results align well with object-perception studies suggesting that aging is associated with decreased weighting of sensory information and greater reliance on top-down influences (Chan et al., 2021; Gilbert and Moran, 2016; Hsu et al., 2021; Trapp et al., 2022). Particularly relevant to the present findings are the studies requiring young and older participants to recognize objects embedded in congruent or incongruent contexts, for example a horse in the woods or in a church (Lai et al., 2020; Rémy et al., 2013). Results suggested that older adults were more affected than the young ones by the object-context congruence, showing an impaired performance in recognizing objects in incongruent contexts. Furthermore, event related potential results (Lai et al., 2020) showed that the components associated with bottom-up sensorial processing (i.e., N1) were altered in the aging group, while those associated with top-down modulation of visual processing and semantic integration (i.e., P2, N400) were preserved. Accordingly, Gilbert and Moran (2016) showed greater feedback connectivity from prefrontal to posterior areas in older compared to young adults during a repetition-priming object naming task, suggesting that aging leads to a more prominent role of top-down signals from prefrontal cortex in driving perception based on contextual expectations and previous experience.

While our behavioral study cannot provide information about the neural underpinnings of the aging-related increase of contextual modulation of action prediction, previous studies (Amoruso et al., 2018, 2023) have documented an important role of prefrontal inputs in driving contextual expectations about action unfolding; indeed, interferential stimulation of the dorsolateral prefrontal cortex hindered any difference in predicting actions embedded in congruent and incongruent contexts. Future studies are needed to show whether and how greater inputs from prefrontal to posterior areas may exert greater influence on action prediction in the aging brain.

### 4.5 Motor imagery and action prediction abilities

Based on theoretical accounts linking motor imagery and forward modeling (Jeannerod, 1994; Miall and Wolpert, 1996), we also explored whether individual differences in motor imagery and action identification might influence participants’ ability to generate predictions of unfolding actions. Specifically, we assessed kinesthetic and visual imagery abilities using the KVIQ and we evaluated action identification level using the BIF. The correlation analyses did not reveal any significant results that may support the relationship between individual differences in motor imagery or action identification and the magnitude of context-based effects on action prediction performance.

Results showed no significant age-related differences in the KVIQ-k score, indicating that the intensity of kinesthetic sensations during motor imagery was comparable across groups. This aligns with previous evidence suggesting that motor imagery is preserved in older adults when imagining familiar actions (Saimpont et al., 2013; Malouin et al., 2010). However, our group of older adults reported more vivid visual representations of imagined movements compared to younger adults, consistent with prior research indicating the recruitment of compensatory visual strategies in aging during motor imagery tasks (Zapparoli et al., 2013, 2016). This may reflect differential engagement of neural networks, as kinesthetic imagery typically activates frontoparietal areas more strongly, while visual imagery recruits visual regions (Hétu et al., 2013; Oldrati et al., 2021; Solodkin et al., 2004; Yang et al., 2021).

Regarding action identification, although the BIF scores did not significantly differ between groups, the slightly higher scores observed in the older group may reflect a subtle tendency to conceptualize actions at a more abstract level. This pattern could indicate reduced embodiment or diminished reliance on sensorimotor representations in aging (Costello and Bloesch, 2017), potentially influencing how observed actions are processed.

### 4.6 Limitations and conclusions

The interpretation of results of this study should consider important limitations. First, although the sample size was estimated to detect a large effect of aging on context-based predictions, it may have been insufficient to account for interindividual variability, limiting generalizability. Similarly, the cross-sectional design did not allow us to capture developmental trends in how aging affects the weighting of kinematic and contextual cues.

Another limitation is the absence of a control task assessing object recognition in context, which prevents us from determining whether the observed age-related differences are specific to motor-related processes or reflect broader perceptual or cognitive changes. Nonetheless, the finding that older adults effectively used contextual cues to enhance prediction accuracy and speed suggests these effects are not solely due to low-level visual processing deficits.

Additionally, the study did not include the kinematic profiling of how the young- and older-adults perform the observed actions, as done in a previous study with young-adult observers (Bianco et al., 2024). Research shows that observers weigh specific kinematic parameters, such as movement speed, grip aperture, and acceleration, differently depending on contextual demands and individual characteristics (Montobbio et al., 2022; Arcuri et al., 2025; Becchio et al., 2024). Since aging may affect the way people move (Kim et al., 2025), we cannot rule out that the difficulty of older-adults in reading out the kinematics of the actions may reflect dissimilarities between actors’ and the observers’ kinematics. Future studies integrating precise kinematic analysis may help clarify which features of an action most strongly influence predictive processing across the lifespan.

In conclusion, we provided evidence that an aging-related decline in the ability to read out early kinematics for action prediction may be compensated by a greater reliance on the contextual scenarios in which actions are embedded. On one hand, this finding corroborates previous studies of reduced perceptual sensitivity to action kinematics in older adults (Diersch et al., 2012; Diersch et al., 2013; Sacheli et al., 2023). On the other hand, it extends to the action perception domain the notion that aging is associated to a greater weighting of contextual expectations to compensate for blurred sensory inputs (Lai et al., 2020; Rémy et al., 2013).

## Data Availability

The datasets presented in this study can be found in online repositories. The names of the repository/repositories and accession number(s) can be found at: https://osf.io/umny6/ Open Science Framework (OSF) repository.
